# Cost-Effectiveness of Sertraline in Primary Care According to Initial Severity and Duration of Depressive Symptoms: Findings from the PANDA RCT

**DOI:** 10.1007/s41669-019-00188-5

**Published:** 2019-11-27

**Authors:** William Hollingworth, Christopher G. Fawsitt, Padraig Dixon, Larisa Duffy, Ricardo Araya, Tim J. Peters, Howard Thom, Nicky J. Welton, Nicola Wiles, Glyn Lewis, the PANDA Team

**Affiliations:** 1 Bristol Medical School, University of Bristol, 5 Tyndall Avenue, Bristol, BS8 1UD UK; 2 Health Information and Quality Authority, City Gate, Mahon, Cork, Ireland; 3 Division of Psychiatry, University College London, 6th Floor Maple House, 149 Tottenham Court Road, London, W1T 7NF UK; 4 Institute of Psychiatry, Psychology and Neuroscience, Kings’ College London, Denmark Hill, London, SE5 8AF UK

## Abstract

**Background:**

Antidepressants are commonly prescribed for depression, but it is unclear whether treatment efficacy depends on severity and duration of symptoms and how prescribing might be targeted cost-effectively.

**Objectives:**

We investigated the cost-effectiveness of the antidepressant sertraline compared with placebo in subgroups defined by severity and duration of depressive symptoms.

**Methods:**

We undertook a cost-effectiveness analysis from the perspective of the NHS and Personal and Social Services (PSS) in the UK alongside the PANDA (What are the indications for Prescribing ANtiDepressants that will leAd to a clinical benefit?) randomised controlled trial (RCT), which compared sertraline with placebo over a 12-week period. Quality of life data were collected at baseline and at 2, 6, and 12 weeks post-randomisation using EQ-5D-5L, from which we calculated quality-adjusted life years (QALYs). Costs (in 2017/18£) were collected using patient records and from resource use questionnaires administered at each follow-up interval. Differences in mean costs and mean QALYs and net monetary benefits were estimated. Our primary analysis used net monetary benefit regressions to identify any interaction between the cost-effectiveness of sertraline and subgroups defined by baseline symptom severity (0–11; 12–19; 20+ on the Clinical Interview Schedule—Revised) and, separately, duration of symptoms (greater or less than 2 years duration). A secondary analysis estimated the cost-effectiveness of sertraline versus placebo, irrespective of duration or severity.

**Results:**

There was no evidence of an association between the baseline severity of depressive symptoms and the cost-effectiveness of sertraline. Compared to patients with low symptom severity, the expected net benefits in patients with moderate symptoms were £24 (95% CI − £280 to £328; *p* value 0.876) and the expected net benefits in patients with high symptom severity were £37 (95% CI − £221 to £296; *p* value 0.776). Patients who had a longer history of depressive symptoms at baseline had lower expected net benefits from sertraline than those with a shorter history; however, the difference was uncertain (− £27 [95% CI − £258 to £204]; *p* value 0.817). In the secondary analysis, patients treated with sertraline had higher expected net benefits (£122 [95% CI £18 to £226]; *p* value 0.101) than those in the placebo group. Sertraline had a high probability (> 95%) of being cost-effective if the health system was willing to pay at least £20,000 per QALY gained.

**Conclusions:**

We found insufficient evidence of a prespecified threshold based on severity or symptom duration that GPs could use to target prescribing to a subgroup of patients where sertraline is most cost-effective. Sertraline is probably a cost-effective treatment for depressive symptoms in UK primary care.

**Trial Registration:**

Controlled Trials ISRCTN Registry, ISRCTN84544741.

**Electronic supplementary material:**

The online version of this article (10.1007/s41669-019-00188-5) contains supplementary material, which is available to authorized users.

## Key Points for Decision Makers

**Table:** 

Sertraline is often prescribed by GPs for symptoms of depression, but it is unclear which patients benefit most.
We found insufficient evidence that either symptom severity or duration could be used by GPs to better target the prescribing of sertraline.
Sertraline is probably a cost-effective treatment for depressive symptoms in UK primary care.

## Introduction

Depression is the leading cause of disability worldwide [1], affecting 4.4% of the world’s population, or 322 million people [2]. Between 2005 and 2015, the number of people living with depression increased by 18.4% [1]. First-line pharmacological treatment of depression is usually with selective serotonin reuptake inhibitor (SSRI) antidepressants, which provide small beneficial effects over placebo [3]. Use of antidepressants has increased dramatically in recent years, particularly in high-income countries. In 2016, 65 million antidepressant items were prescribed in England, more than double the number prescribed ten years earlier (31 million) [4]. The associated healthcare costs of increased use of antidepressants are considerable, despite the introduction of cheaper generic SSRI equivalents. In 2016, primary care prescriptions of antidepressants in England cost the National Health Service (NHS) £266.6 million [4].

Depression is typically managed by general practitioners (GPs), with the exception of a small proportion of cases that require specialist mental or social care services [5]. Management options include active monitoring, low-intensity psychosocial interventions, group cognitive behavioural therapy and drug treatment [6]. The decision to prescribe is generally based on clinical judgement of patients’ symptoms and not on the results of standardised scales or questionnaires. Patients may be considered mildly, moderately or severely depressed, depending on their associated symptoms [5, 7]; however, it is unclear which patient groups are most likely to benefit from treatment with SSRIs. Patients with mild depressive symptoms are commonly prescribed antidepressants despite not meeting International Classification of Diseases (ICD-10) criteria for depression [5, 8], and there is limited evidence to support the use of antidepressants in this population [9]. It has been proposed that patients with more severe symptoms may benefit more from antidepressants, although the evidence is not clear-cut [10–14]. Patients with dysthymia—depression that is present for two or more years but does not meet diagnostic criteria [15]—might also benefit more from antidepressants. Previous studies were not designed to explore the cost-effectiveness of antidepressants by severity and duration of depression.

We undertook a large pragmatic randomised controlled trial (the PANDA RCT—What are the indications for Prescribing ANtiDepressants that will leAd to a clinical benefit?) to investigate the clinical effectiveness of a commonly prescribed SSRI, sertraline, versus placebo, and to explore the influence of severity and duration of depressive symptoms on response to sertraline compared with placebo in people presenting to primary care with depression. The results of the RCT are detailed in Lewis et al. [16]. In this paper, we estimated the cost-effectiveness of sertraline in comparison with placebo at the end of 12 weeks of follow-up in relation to the baseline severity and duration of depressive symptoms. A secondary analysis estimated the cost-effectiveness of sertraline in comparison with placebo in all patients, irrespective of severity or duration.

## Methods

The published trial protocol provides full details of eligibility criteria, recruitment, randomisation, treatment regimens and assessment methods [17]. In brief, PANDA is a randomised, double-blind, placebo-controlled trial in which participants are individually randomised to sertraline or placebo. Eligible participants aged between 18 and 74 were identified in primary care with depression or low mood during the past 2 years and had not received antidepressant or anti-anxiety medication in the previous 8 weeks. Trial participants were recruited from 179 primary care surgeries in four UK sites. Participants were randomised to receive either sertraline or matching placebo, starting at 50 mg daily for 1 week, increasing to 100 mg daily for up to 11 weeks (with the option of increasing to 150 mg if required). Sertraline is a cheap off-patent medication (approximately £0.80–1.00 per 28 tablets) [18]. The primary outcome was depressive symptoms measured by the Patient Health Questionnaire 9 (PHQ-9) at 6 weeks postrandomisation. The clinical effectiveness findings of the PANDA trial have been reported in detail [16], and are briefly summarised here in the “Results” section.

The primary economic analysis took an NHS and personal social services (PSS) perspective. A secondary analysis was undertaken from the perspective of individual patients, accounting for costs such as expenditure on private health care. We also considered the cost to society of work absences. The time horizon for the economic analysis was up to 12 weeks post-randomisation, reflecting the duration of follow-up in the trial. As the follow-up period does not extend beyond one year, discounting of costs and benefits was not applied. The analysis plan was agreed with the trial steering committee and was deposited in UCL Discovery [19].

### Estimating Benefits

The primary economic outcome measure was quality-adjusted life years (QALYs) derived from utility scores, obtained using the EQ-5D-5L quality of life instrument [20]. EQ-5D-5L questionnaires were completed by participants prior to randomisation (baseline) and at 2, 6 and 12 weeks post-randomisation. Baseline and research follow-up assessments took place at the participant’s home, general practice, or at university premises. Utility scores were derived from responses to the EQ-5D-5L using valuations obtained from an English population [21]. We used linear interpolation of responses at baseline and at 2, 6 and 12 weeks to estimate QALYs over the 12-week period, adjusting for baseline EQ-5D-5L scores in regression analyses as recommended in the literature [22].

### Estimating Costs

For the primary analysis, data were collected on health service use, including primary care consultations, sertraline, other prescribed medication, hospital admission, outpatient attendance, emergency department attendance and community-based care. For secondary analyses, we also included productivity losses due to mental health problems and personal costs (e.g. payments for additional care). Primary care consultations were captured through electronic downloads of GP records. Manual data extraction was used as a backup if GP records did not support automatic downloads. NHS secondary care, community care, care from social services, time off work and patient personal resource use during trial follow-up was captured using patient-reported questionnaires completed at 2, 6 and 12 weeks.

The cost of medications was estimated from the British National Formulary [23]. Community and primary care costs were based on national estimates [24]. Codes for Healthcare Resource Groups (groups of events that have been judged to consume similar levels of resources) were assigned to secondary care contacts and were costed based on aggregate national reference costs where available (for example, ultrasound) [25]. Productivity costs were estimated based on national average weekly earnings stratified by sex [26]. All costs are reported for the financial year 2017/18 in pounds sterling (£) and adjusted for inflation using the Hospital and Community Health Services Index where necessary [24].

### Cost-Effectiveness Analysis

A cost-effectiveness analysis was conducted using the intention-to-treat principle, comparing the two groups as randomised and including all patients in the analysis. This analysis estimated the cost-effectiveness of sertraline and how the net monetary benefit (NMB) of sertraline varies with baseline severity of depression and with symptom duration. NMB is a summary statistic used to present cost-effectiveness results. It estimates the value of an intervention in monetary terms using a predefined willingness to pay threshold for a unit of benefit, typically QALY. An intervention is said to be cost-effective if the NMB is positive. The NMB is recommended for use in identifying heterogeneity in cost-effectiveness between subgroups as, unlike the incremental cost-effectiveness ratio (ICER) that was specified in the trial protocol, the NMB is a linear combination of individual patient costs and QALYs that facilitates regression analysis using subgroup interaction terms [27].

Data cleaning was undertaken prior to the unblinding of the economic researcher to the study groups. This involved the correction of obvious ‘free text’ response errors (e.g. misspelt drug names) and group coding of similar resource items to enable unit costing. Multiple imputation was subsequently undertaken for missing data. The primary analysis was based on imputed datasets and included all participants [28, 29]. We implemented multiple imputation by chained equations on data that were assumed missing at random. The imputation model was stratified by trial arm and included complete demographic data (e.g. age, ethnicity, financial stability, marital status) alongside clinical outcome variables (on severity and duration of depressive symptoms) at baseline, utility score at baseline, cost and utility variables with missing data, and site practice (i.e. London, Bristol, Liverpool, York). We imputed costs at the aggregate level (e.g. total NHS and PSS costs). We imputed utility data for each follow-up period and used these to generate QALY estimates. The number of imputations (*n* = 40) was selected to be greater than the proportion of missing data, as per White et al. [30]. A complete case analysis was also undertaken for comparative purposes.

We estimated the incremental difference in mean total costs and mean QALYs between the two arms of the trial and 95% confidence intervals using ordinary least squares regression. In the primary analysis, cost and QALY data were combined to calculate a NMB statistic [31] from the NHS and PSS perspective. For each individual *i*, the NMB statistic is NMB_*i*_ = *λE*_*i*_ − *C*_*i*_, where *C*_*i*_ is the cost of care for that individual, *E*_*i*_ is the QALYs of that individual, and *λ* is the cost-effectiveness threshold (i.e. the amount society is willing to pay for a QALY). In all analyses, we used the commonly used NICE threshold values of £20,000 and £30,000 per QALY. We estimated NMB regressions using interactions between the treatment indicator and baseline severity (model 1) and in a separate model between the treatment indicator and symptom duration (model 2) to explore whether sertraline was differentially more cost-effective across subgroups defined by symptom severity or duration. Three severity strata (0–11; 12–19; 20+ on the Clinical Interview Schedule—Revised (CIS-R) at baseline) [32] and two duration strata (less than 2 years or 2 years or more) were prespecified in the trial protocol [17]. The CIS-R is a widely used diagnostic assessment tool for mental health conditions. The CIS-R score indicates the severity of psychological symptoms. A positive regression coefficient on the interaction term between the treatment group and patient subgroup indicates that sertraline is potentially more cost-effective in that subgroup compared with the reference subgroup.

In a prespecified secondary analysis, we estimated the cost-effectiveness of sertraline versus placebo (model 3), irrespective of baseline severity and symptom duration. All regression models adjusted for baseline utility scores and study centre as a random effect. Uncertainty in the point estimates of NMB was quantified using 95% confidence intervals estimated from the regressions. We calculated cost-effectiveness acceptability curves (CEACs) [33] to probe the uncertainty in the optimal treatment for various different cost-effectiveness thresholds.

In a post-hoc sensitivity analysis, we examined the impact of excluding secondary care and prescriptions extracted from medical records that were judged to be not clearly related to the treatment of depression (Supplementary Table 1 in the Electronic supplementary material, ESM). We also conducted a further sensitivity analysis in which we removed all secondary care costs from total NHS and PSS costs. This assessed whether our findings were robust to infrequent but expensive hospitalisations that might differ between arms by chance.

The economic evaluation followed best practice guidelines, as outlined by Husereau et al. [34] and detailed in Supplementary Table 2 in the ESM. Stata software (version 15) [35] was used for all analyses.

## Results

### Patient Characteristics and Trial Outcomes

We randomised 655 patients, 326 to sertraline and 329 to placebo. Two patients were missing substantial proportions of the baseline assessment and were excluded, leaving 653 for analyses. 505 (77.3%) patients had complete EQ-5D-5L responses at all four assessment points. A smaller proportion (381/653; 58.3%) had complete resource use data available from primary care notes and self-reported assessments. Follow-up rates were similar in both arms at all time points (Supplementary Fig. 1 in the ESM). At recruitment, the mean age was 39.7 years (SD 14.96), 59% (*n* = 384) were female, and the mean PHQ-9 score was 12.0 (SD 5.8) [16]. Table 1 provides details of baseline PHQ-9 scores and EQ-5D-5L scores across the subgroups defined by severity and duration. At baseline, patients with severe or prolonged depressive symptoms (≥ 2 years) had worse EQ-5D-5L and PHQ-9 scores. As previously reported, there was a 5% relative reduction (adjusted proportional change 0.95 [95% CI 0.85–1.07]) in PHQ-9 depressive symptom scores in the sertraline group at 6 weeks, but the confidence interval did not exclude the possibility of no treatment effect [16]. There was stronger evidence for a treatment effect of sertraline on PHQ-9 score at 12 weeks (adjusted proportional change 0.87 [95% CI 0.79–0.97]) [16]. In the sections below, we first report the cost-effectiveness of sertraline versus placebo (secondary analysis) and then explore whether sertraline was differentially more cost-effective across subgroups defined by symptom severity or duration (primary analyses).

**Table 1:** Baseline health-related quality of life in subgroups defined by depressive symptom severity and duration

	Sertraline (*n* = 324)	Placebo (*n* = 329)	Overall (*n* = 653)
	Mean (SE)	Mean (SE)	Mean (SE)
Severity subgroups			
EQ5D-5L			
Mild (*n* = 129)	0.816 (0.014)	0.796 (0.017)	0.806 (0.011)
Moderate (*n* = 173)	0.769 (0.016)	0.800 (0.012)	0.785 (0.010)
Severe (*n* = 349)	0.652 (0.016)	0.653 (0.016)	0.653 (0.011)
PHQ-9			
Mild (*n* = 128)	5.657 (0.410)	6.049 (0.548)	5.844 (0.337)
Moderate (*n* = 173)	9.500 (0.432)	10.258 (0.420)	9.890 (0.302)
Severe (*n* = 350)	15.308 (0.370)	15.275 (0.349)	15.291 (0.254)
Duration subgroups			
EQ5D-5L			
< 2 years (*n* = 439)	0.729 (0.012)	0.743 (0.012)	0.736 (0.009)
≥ 2 years (*n* = 212)	0.690 (0.020)	0.673 (0.020)	0.681 (0.014)
PHQ-9			
< 2 years (*n* = 438)	11.408 (0.400)	11.550 (0.388)	11.479 (0.278)
≥ 2 years (*n* = 213)	12.600 (0.567)	13.519 (0.521)	13.066 (0.385)

### Cost-Effectiveness (Sertraline Versus Placebo)

In the imputed dataset, total NHS and PSS costs were lower in the sertraline group (£154) than in the placebo group (£177), but the difference was small and there was no evidence of a treatment effect on costs (difference − £22 [95% CI − £87 to £42]; *p* value 0.490) (Table 2). Sensitivity analyses excluding secondary care costs and costs not clearly associated with mental health care supported this finding, as did the complete case analysis. There was little difference in patient costs or time off work costs (i.e. productivity losses) between groups. The cost of sertraline in the intervention group over the 12 weeks of the trial was minimal. There were no large or consistent differences in the cost of other medications, primary care consultations, community-based care or hospital care between the sertraline or placebo groups during the 12-week follow-up.

**Table 2:** Summary costs

	Sertraline (*n* = 324)	Placebo (*n* = 329)	Mean difference (*n* = 653)	
	Mean (SE)^c^	Mean (SE)^c^	Mean (95% CI)	*p* value
Imputed				
Total NHS costs^a^	£154.01 (£18.97)	£176.5 (£25.71)	£− 22.48 (− £86.61 to £41.64)	0.490
Total NHS mental-health-related costs^b^	£35.26 (£7.96)	£55.12 (£12.11)	£− 19.86 (− £49.16 to £9.44)	0.182
Total NHS costs, excluding secondary care	£98.39 (£14.92)	£121.43 (£15.58)	£− 23.04 (− £65.19 to £19.12)	0.283
Total productivity losses	£134.38 (£32.27)	£120.58 (£25.93)	£13.8 (− £68.03 to £95.63)	0.740
Total patient costs	£4.73 (£2.55)	£7.93 (£2.68)	£− 3.2 (− £10.46 to £4.06)	0.386

	Sertraline (*n* = 174)	Placebo (*n* = 207)	Mean difference (*n* = 381)	
	Mean (SD)	Mean (SD)	Mean (95% CI)	*p* value
Complete cases				
Total NHS costs^a^	£169.34 (£351.17)	£188.42 (£423.05)	− £19.08 (− £98.33 to £60.16)	0.636
Total NHS mental-health-related costs^b^	£28.98 (£93.65)	£50.92 (£167.33)	− £21.94 (− £49.98 to £6.1)	0.125
Total NHS costs, excluding secondary care	£112.18 (£303.14)	£124.36 (£274.77)	− £12.18 (− £70.44 to £46.07)	0.681
Total productivity losses	£118.22 (£484.05)	£69.61 (£246.46)	£48.6 (− £27.05 to £124.26)	0.207
Total patient costs	£5.41 (£41.52)	£10.83 (£47.24)	− £5.42 (− £14.46 to £3.62)	0.239
Disaggregated costs (complete cases)				
Sertraline	£3.81 (£0.62)	£0 (£0)	£3.81 (£3.72 to £3.89)	0.000
Primary care consultations				
GP consultations	£26.16 (£44.48)	£27.53 (£39.55)	− £1.37 (− £9.84 to £7.1)	0.750
Nurse consultations	£3.93 (£13)	£5.14 (£11.3)	− £1.21 (− £3.66 to £1.24)	0.332
GP telephone calls	£3.23 (£8.55)	£2.72 (£8.69)	£0.52 (− £1.23 to £2.26)	0.562
Nurse telephone calls	£0.5 (£2.43)	£0.65 (£3.28)	− £0.15 (− £0.74 to £0.44)	0.620
Medications				
All	£49.61 (£272.99)	£37.64 (£215.99)	£11.97 (− £37.31 to £61.25)	0.633
Mental health	£0.23 (£1.46)	£0.23 (£2.2)	£0 (− £0.39 to £0.38)	0.995
Secondary care				
Inpatient care	£3.55 (£46.78)	£20.71 (£258.4)	− £17.16 (− £56.21 to £21.89)	0.388
Outpatient care	£35.83 (£93.22)	£33.39 (£102.76)	£2.44 (− £17.49 to £22.36)	0.810
Accident and emergency	£17.78 (£85.67)	£9.97 (£42.4)	£7.82 (− £5.48 to £21.12)	0.249
Community-based care				
Community care	£23.6 (£90.59)	£47.99 (£164.74)	− £24.39 (− £51.9 to £3.11)	0.082
Home visits	£1.33 (£14.68)	£2.45 (£30.18)	− £1.12 (− £6.05 to £3.81)	0.655
Additional help	£0.02 (£0.23)	£0.25 (£2.88)	− £0.23 (− £0.66 to £0.2)	0.296

*CI* confidence interval, *NHS* National Health Service, *PSS* Personal and Social Services, *SD* standard deviation, *SE* standard error

^a^Primary, secondary, and community-based care

^b^Mental-health-related medication and community care costs

^c^Standard errors reported here are due to imputed datasets

**Fig. 1:**
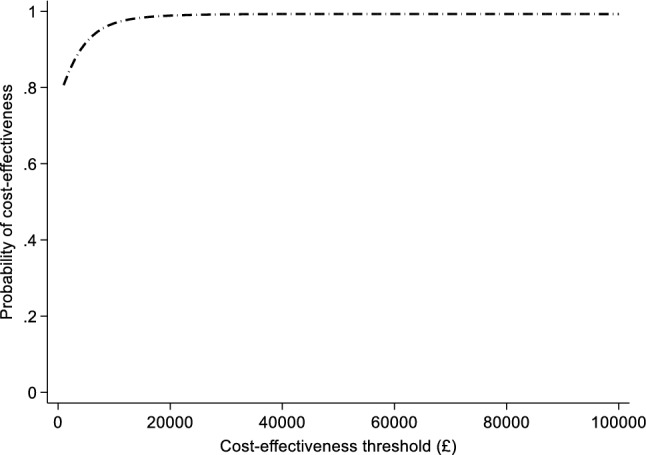
Cost effectiveness acceptability curve of sertraline versus placebo (imputed)

Over the 12-week period, QALYs were marginally higher in the sertraline group (0.182) than the placebo group (0.177), but there was no strong evidence of a treatment effect using the imputed dataset (QALY difference 0.005 [95% CI − 0.003 to 0.012]; *p* value 0.150) (Table 3). However, preference-based quality of life scores measured by the EQ-5D-5L increased (i.e. improved) in both groups from baseline to 12 weeks. There was some evidence that sertraline was more cost-effective than placebo at a threshold of £20,000 (incremental NMB £122 [95% CI £18 to £226]; *p* value 0.022) and £30,000 (incremental NMB £171 [95% CI £33 to £310]; *p* value 0.016) per QALY, respectively (Table 4, model 3); sertraline had a high probability (> 95%) of being cost-effective if the health system is willing to pay more than £20,000 per QALY gained (Fig. 1).

**Table 3:** Utility scores and QALYs

	Sertraline (*n* = 324)	Placebo (*n* = 329)	Mean difference (*n* = 653)	
	Mean (SE)	Mean (SE)	Mean (95% CI)	*p* value
Imputed				
QALYs	0.182 (0.002)	0.177 (0.002)	0.005 (− 0.003 to 0.012)	0.150

	Sertraline (*n* = 248)	Placebo (*n* = 257)	Mean difference (*n* = 505)	
	Mean (SD)	Mean (SD)	Mean (95% CI)	*p* value
Complete cases				
Baseline	0.732 (0.172)	0.725 (0.188)	0.008 (− 0.024 to 0.039)	0.638
2 weeks	0.774 (0.164)	0.756 (0.176)	0.018 (− 0.012 to 0.048)	0.236
6 weeks	0.807 (0.180)	0.776 (0.185)	0.031 (− 0.001 to 0.063)	0.054
12 weeks	0.815 (0.184)	0.790 (0.185)	0.025 (− 0.007 to 0.057)	0.128
QALYs	0.183 (0.034)	0.178 (0.037)	0.006 (− 0.002 to 0.013)	0.078

*CI* confidence interval, *QALYs* quality-adjusted life years, *SD* standard deviation, *SE* standard error

**Table 4:** Incremental net monetary benefit results (imputed)

	£20,000 WTP		£30,000 WTP	
	INMB (95% CI)	*p* value	INMB (95% CI)	*p* value
Model 1 (*n* = 650)				
Sertraline	£91.94 (− £123.17 to £307.06)	0.401	£129.23 (− £150.48 to £408.94)	0.364
Severity				
Low	Reference		Reference	
Moderate	− £33.55 (− £275.23 to £208.13)	0.785	− £56.28 (− £354.6 to £242.04)	0.711
High	− £58.02 (− £337.46 to £221.42)	0.683	− £95.19 (− £457.35 to £266.98)	0.605
Sertraline × severity				
Low	Reference		Reference	
Moderate	£24.18 (− £279.96 to £328.31)	0.876	£47.23 (− £341.78 to £436.24)	0.811
High	£37.44 (− £221.35 to £296.24)	0.776	£44.46 (− £304.27 to £393.19)	0.802
Model 2 (*n* = 650)				
Sertraline	£127.41 (− £2.25 to £257.08)	0.054	£177.96 (£9.14 to £346.78)	0.039
Duration (> 2 years)	£26.61 (− £125.95 to £179.18)	0.732	£10.71 (− £196.18 to £217.61)	0.919
Sertraline × duration (> 2 years)	− £27.14 (− £258.13 to £203.86)	0.817	− £37.39 (− £360.59 to £285.81)	0.820
Model 3 (*n* = 653)				
Sertraline vs placebo	£121.83 (£17.79 to £225.87)	0.022	£171.12 (£32.68 to £309.55)	0.016

Model 1: interaction model between sertraline and baseline severity, adjusted for severity, duration, baseline utility and site practice

Model 2: interaction model between sertraline and duration, adjusted for duration, severity, baseline utility and site practice

Model 3: non-interaction model comparing sertraline vs placebo, adjusted for baseline utility and site practice

*CI* confidence interval, *INMB* incremental net monetary benefit

### Cost-Effectiveness (in Subgroups Defined by Symptom Severity and Duration)

Participants with higher symptom severity reported lower utility scores during trial follow-up (Table 5), although several patients reported high QALY scores despite high baseline symptom severity (Supplementary Fig. 2 in the ESM). QALYs were slightly higher in the sertraline group in all three depressive symptom severity strata, but in each case the confidence interval included zero (Table 5). There were no consistent differences in the incremental costs of sertraline across all three symptom strata, and confidence intervals were wide (Table 5).

**Table 5:** Costs and outcomes in subgroups defined by depressive symptom severity (complete cases except where stated)

	Sertraline	Placebo	Difference
	Mean (SD)	Mean (SD)	Mean (95% CI)
Mild (*n* = 103)			
Baseline utility score	0.815 (0.121)	0.806 (0.121)	0.009 (− 0.038 to 0.057)
2 weeks utility score	0.842 (0.112)	0.825 (0.139)	0.016 (− 0.032 to 0.067)
6 weeks utility score	0.874 (0.138)	0.843 (0.131)	0.030 (− 0.022 to 0.083)
12 weeks utility score	0.893 (0.123)	0.860 (0.133)	0.033 (− 0.017 to 0.083)
QALYs (imputed, *n* = 129)	0.199 (0.003 SE)	0.192 (0.004 SE)	0.004 (− 0.004 to 0.012)
Total NHS costs (imputed, *n* = 129)	£163.22 (£36.46 SE)	£188.65 (£58.71 SE)	− £18.92 (− £154.15 to £116.31)
Incremental NMB^a^ (imputed, *n* = 129)			£101.83 (− £113.68 to £317.34)
Moderate (*n* = 137)			
Baseline utility score	0.776 (0.134)	0.792 (0.117)	− 0.017 (− 0.059 to 0.026)
2 weeks utility score	0.814 (0.163)	0.808 (0.101)	0.006 (− 0.040 to 0.052)
6 weeks utility score	0.835 (0.183)	0.828 (0.132)	0.007 (− 0.046 to 0.061)
12 weeks utility score	0.861 (0.163)	0.810 (0.157)	0.051 (− 0.003 to 0.105)
QALYs (imputed, *n* = 173)	0.191 (0.004 SE)	0.189 (0.003 SE)	0.007 (0 to 0.014)
Total NHS costs (imputed, *n* = 173)	£175.5 (£50.35 SE)	£160.03 (£56.17 SE)	£3.65 (− £145.04 to £152.35)
Incremental NMB^a^ (imputed, *n* = 173)			£134.56 (− £69.39 to £338.51)
Severe (*n* = 265)			
Baseline utility score	0.676 (0.187)	0.660 (0.213)	0.016 (− 0.032 to 0.065)
2 weeks utility score	0.726 (0.168)	0.704 (0.202)	0.022 (− 0.023 to 0.067)
6 weeks utility score	0.766 (0.183)	0.724 (0.209)	0.041 (− 0.006 to 0.089)
12 weeks utility score	0.760 (0.198)	0.754 (0.206)	0.006 (− 0.043 to 0.055)
QALYs (imputed, *n* = 351)	0.171 (0.003 SE)	0.166 (0.003 SE)	0.005 (− 0.002 to 0.011)
Total NHS costs (imputed, *n* = 351)	£140.02 (£18.59 SE)	£180.5 (£28.41 SE)	− £40.7 (− £108.63 to £27.24)
Incremental NMB^a^ (imputed, *n* = 351)			£131.18 (− £18.49 to £280.86)

^a^At £20,000 per QALY threshold

**Fig. 2:**
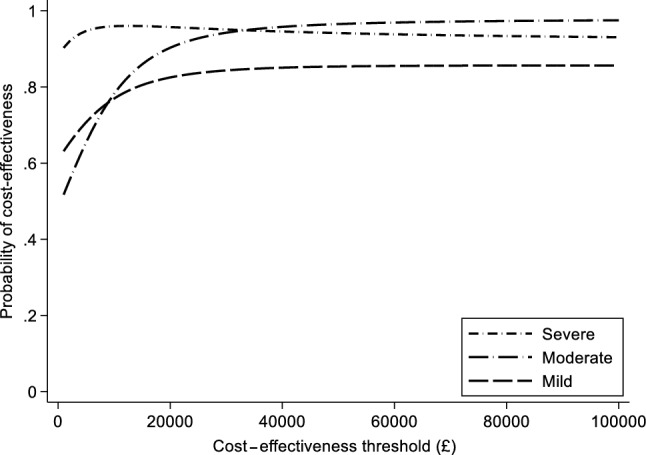
Cost-effectiveness acceptability curves of sertraline versus placebo in patients with different levels of severity of depression (imputed)

There was no evidence of an association between the baseline severity of depressive symptoms and the cost-effectiveness of sertraline (Table 4, model 1). Compared to patients with low symptom severity at baseline, patients with moderate symptoms (difference in incremental NMB £24 [95% CI − £280 to £328]; *p* value 0.876) and patients with high symptom severity had higher incremental net benefits of sertraline (difference in incremental NMB £37 [95% CI − £221 to £296]; *p* value 0.776). Similar findings were observed at the £30,000 per QALY threshold. Sertraline was probably (*p* > 0.7) cost-effective in all three severity subgroups at the £20,000 per QALY threshold (Fig. 2).

**Fig. 3:**
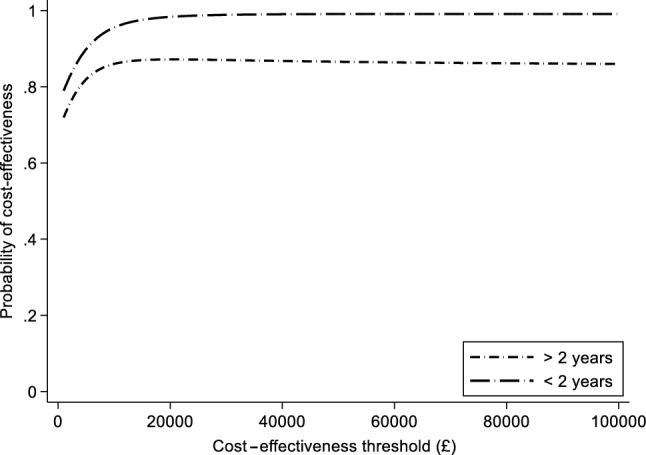
Cost-effectiveness acceptability curves of sertraline versus placebo in patients with different levels of symptom duration (imputed)

There was little obvious association between symptom duration and QALYs or costs (Supplementary Fig. 2 in the ESM). QALYs were slightly higher in the sertraline group in both strata of symptom duration, but the confidence intervals approached zero (Table 6). The NHS costs were lower in the sertraline group in both strata, but the confidence intervals were wide and included zero (Table 6). Patients who had a longer history of depressive symptoms at baseline (Table 4, model 2) had lower incremental net benefits from sertraline than those with a shorter history; however, the confidence intervals were wide (difference in incremental net benefit − £27 [95% CI − £258 to £204] at the £20,000 per QALY threshold; *p* value 0.817). The probability that sertraline is cost-effective in those with longer symptom duration exceeds 0.8 (Fig. 3).

**Table 6:** Costs and outcomes in subgroups defined by symptom duration (complete cases except where stated)

	Sertraline	Placebo	Difference
	Mean (SD)	Mean (SD)	Mean (95% CI)
< 2 years (*n* = 342)			
Baseline utility score	0.732 (0.182)	0.734 (0.182)	− 0.003 (− 0.041 to 0.036)
2 weeks utility score	0.774 (0.176)	0.764 (0.170)	0.011 (− 0.026 to 0.048)
6 weeks utility score	0.813 (0.194)	0.782 (0.191)	0.031 (− 0.010 to 0.072)
12 weeks utility score	0.837 (0.170)	0.808 (0.181)	0.029 (− 0.008 to 0.067)
QALYs (imputed, *n* = 439)	0.185 (0.003 SE)	0.181 (0.002 SE)	0.006 (0.001 to 0.01)
Total NHS costs (imputed, *n* = 439)	£168.6 (£24.76 SE)	£192.2 (£33.33 SE)	− £23.6 (− £106.11 to £58.92)
Incremental NMB (imputed, *n* = 439)			£139.72 (£11.39 to £268.06)
≥ 2 years (*n* = 163)			
Baseline utility score	0.733 (0.151)	0.703 (0.200)	0.030 (− 0.024 to 0.085)
2 weeks utility score	0.774 (0.138)	0.740 (0.189)	0.034 (− 0.017 to 0.085)
6 weeks utility score	0.796 (0.147)	0.763 (0.172)	0.033 (− 0.017 to 0.082)
12 weeks utility score	0.771 (0.204)	0.750 (0.189)	0.021 (− 0.040 to 0.082)
QALYs (imputed, *n* = 214)	0.175 (0.004 SE)	0.169 (0.004 SE)	0.005 (− 0.004 to 0.013)
Total NHS costs (imputed, *n* = 214)	£124.02 (£21.63 SE)	£144.37 (£31.65 SE)	− £17.66 (− £92.59 to £57.27)
Incremental NMB (imputed, *n* = 214)			£107.95 (− £79.84 to £295.74)

### Sensitivity Analyses

Restricting the analysis to patients with complete data did not change the conclusions (Supplementary Table 3 in the ESM). The results remained unchanged in sensitivity analyses in which secondary care costs and costs not clearly associated with mental health care were excluded (Supplementary Tables 4 and 5 in the ESM).

## Discussion

### Key Findings

We found insufficient evidence that variation in the cost-effectiveness of sertraline according to severity or symptom duration at prespecified thresholds could be used by GPs to target prescribing to subgroups of patients. There was no evidence of a substantial treatment effect of sertraline on quality of life as measured by the EQ-5D-5L. However, sertraline is a cheap intervention that had a high probability of being cost-effective compared with placebo, on average, across primary care patients with depression or low mood.

### Strengths and Weaknesses

The PANDA trial is a large publicly funded, placebo-controlled trial of an antidepressant. We used pragmatic eligibility criteria to recruit participants where the GP was uncertain about the benefit of an SSRI, which resulted in randomised patients ranging from those with very few to those with severe depressive symptoms. The wide spectrum of symptom severity and duration increased the likelihood of identifying subgroups where sertraline was more cost-effective. The PANDA trial had a relatively short follow-up period and found no strong evidence that sertraline reduced depressive symptoms measured by the primary outcome (PHQ-9) at 6 weeks [16]. While there was evidence that sertraline reduced depressive symptoms by 12 weeks and reduced anxiety at 6 and 12 weeks, there was no evidence that symptom severity or duration affected response to either depressive or anxious symptoms [16]. Adverse events were rare (four adverse events for sertraline and three for placebo) [16]. In this paper, we have demonstrated that utility scores based on the EQ-5D-5L were marginally higher in the sertraline group at 6 and 12 weeks, although we cannot definitively exclude no treatment effect. We used the newer five-level version of the EQ-5D, which was developed to be more sensitive to changes in health status. However, it is possible that subgroups where sertraline was more cost-effective might have become more evident with longer follow-up or a more sensitive preference-based outcome measure.

Although the PANDA trial is, to our knowledge, the largest trial of an antidepressant to explore differential cost-effectiveness across prespecified subgroups, it was not designed to provide high statistical power on the economic outcome (NMB). Cost-effectiveness outcomes are often measured with less precision than clinical outcomes due to skewed cost distributions (through rare high-cost events) and a higher prevalence of missing data (as cost and QALY data are cumulative measures requiring complete data at every follow-up point). Approximately 40% of patients had at least one missing data point for resource use in the PANDA trial. This proportion is not unusual in primary care trials recruiting patients with depressive symptoms [36]. We assumed data were missing at random and used multiple imputation methods to minimise the risk of bias due to missing data. Nevertheless, missing data reduce the precision of our estimates and decrease the statistical power to identify differential cost-effectiveness across subgroups. We also analysed the two subgroup variables of severity and duration separately, but this neglects any correlation between them (e.g. in those for whom severity is related to duration).

We prespecified symptom severity and duration thresholds in order to reduce the risk of a type 1 error, which could occur if multiple tests are used to compare subgroups defined by a large number of different severity and duration threshold values. However, the prespecified thresholds are essentially arbitrary values with “sharp” cut-off points, and it is possible that other threshold values might have better differentiated between subgroups where sertraline is more or less cost-effective. The PANDA trial only explored one of a large number of potential treatment options for primary care patients with depressive symptoms. The cost-effectiveness of other options, such as cognitive behavioural therapy, might also plausibly vary with symptom severity and duration. The expansion of access to psychological therapy increases the number of treatment options available and also the complexity of selecting the optimal treatment for patient subgroups [37].

### Comparison with Other Studies

Guidelines for conducting economic evaluations alongside RCTs emphasise the value of subgroup analyses for decision makers and the importance of subgroup prespecification to minimise false-positive findings [38]. The National Institute of Health and Care Excellence (NICE) encourages estimates of the interaction between the treatment effect and prespecified, clinically plausible subgroups to identify patient characteristics that increase or decrease the cost-effectiveness of the intervention [39]. However, NICE and other method guides [38] do not specify how cost-effectiveness subgroup analyses should be conducted or interpreted. Further methodological guidance is needed in this area. The decision-making approach, focussing on mean net benefits irrespective of statistical significance, could be applied to subgroup analyses [40], and would probably lead to coverage decisions based on a larger number of subgroups.

NICE note that not all patient subgroups (for example those defined by socioeconomic status) will provide an equitable basis for policy making, even if they are strongly associated with the intervention net benefit. The appropriate use of subgroups can increase population health by targeting spending at those patients where health gains are highest and/or opportunity costs are lowest [41]. In order to achieve this, subgroups need to be defined by characteristics that can be easily and objectively identified in clinical practice; in principle the severity and duration of depressive symptoms fit these criteria. Given the high cost and clinical uncertainty surrounding the appropriate prescribing of antidepressants in primary care, it is surprising that there is not a larger literature identifying subgroups where SSRIs are most (and least) cost-effective.

Previous work based on the THREAD RCT explored how the cost-effectiveness of an SSRI and supportive care versus supportive care alone in primary care patients with new episodes of mild to moderate depression might vary by patient subgroup [42, 43]. That work identified an association between previous episodes of depression and the costs and QALY outcomes of treatment. Based on a comparison of CEACs by subgroup, they concluded that having no previous episode of depression was associated with a higher probability of the SSRI being cost-effective. Our findings, based on a larger sample size and a prespecified hypothesis, provide limited support for this finding. We found that while the incremental net benefit of sertraline was lower in patients with a longer duration of symptoms, the confidence intervals were wide and included zero (i.e. no interaction between duration and cost-effectiveness).

### Implications for Practice and Research

It remains plausible that the cost-effectiveness of SSRIs is related to the duration of symptoms, but our data do not provide sufficient evidence that a threshold symptom duration of 2 years could be used to target prescribing. Individual patient data meta-analysis of SSRI RCTs (e.g. PANDA and THREAD) with economic evaluations could increase the power to detect meaningful subgroups. In practice, though, the lack of homogeneity between these trials (for example the use of a placebo control and the method used to measure QALYs) may limit the viability of this approach. This could improve with increased data sharing and the adoption of core cost and outcome sets in future trials.

Our findings demonstrate that sertraline is probably cost-effective relative to placebo on average across the broad range of depressive symptom severities included in the PANDA trial. However, individual treatment decisions must assess the potential benefits and harms of sertraline. Caution and careful treatment monitoring is particularly required in young adults, where evidence suggests an increased risk of suicidal ideation with SSRI use [44, 45]. There was insufficient evidence that the use of sertraline was differentially cost-effective in any of the three severity subgroups explored in the PANDA economic analysis.

The relatively small differences in cost and outcomes observed between the sertraline and placebo groups will probably continue to make it difficult to identify subgroups where SSRI prescribing is markedly more cost-effective. Subgroup analyses based on economic evaluation may be more likely to be of value to policy makers in situations where the cost/efficacy/safety trade-offs are higher and where net benefit is more strongly associated with subgroup characteristics. In these cases, statistical power will be improved, although large sample sizes will still be required to identify interactions with baseline characteristics.

## Conclusions

There is currently insufficient evidence that the cost-effectiveness of sertraline differs between subgroups defined by symptom severity and duration. Given this, it is likely that clinical judgement and patient preferences will continue to play the predominant role in the initiation of SSRI prescribing.

## Electronic supplementary material

Below is the link to the electronic supplementary material.

Supplementary material 1 (DOCX 682 kb)

## Data Availability

The datasets generated and analysed during the current study are available from the corresponding author (WH) on reasonable request. To gain access, researchers will need to sign a data access agreement with the study sponsor (University College London, London, UK).
