# Supplementary material for: Cost-Effectiveness of Sertraline in Primary Care According to Initial Severity and Duration of Depressive Symptoms: Findings from the PANDA RCT
Source: Pharmacoecon Open. 2019 Nov 27;4(3):427–38. doi: 10.1007/s41669-019-00188-5 (PMC7426336; doi:10.1007/s41669-019-00188-5)
Supplement: Supplementary file 1 — Supplementary material 1 (DOCX 682 kb) [file 41669_2019_188_MOESM1_ESM.docx]

**Supplementary material**

**Supplementary table 1** Services/medications judged to be clearly associated with mental health

| Hospital stays ^1^ | Due to mental breakdown |
| --- | --- |
|  | Attempted suicide |
| A&E visits ^1^ | Anxiety attack |
|  | Attempted overdose |
|  | Suicidal thoughts or intent |
| Hospital outpatient visits ^1^ | Depression |
|  | Suicidal ideation |
| Community care ^1^ | Counselling |
|  | Face-to-face cognitive behavioural therapy (CBT) |
|  | Computer-based cognitive behavioural therapy (CBT) |
|  | Mental health clinic visits |
| Medications ^2^ | Amitriptyline |
|  | Citalopram |
|  | Diazepam |
|  | Duloxetine |
|  | Fluoxetine |
|  | Gabapentin |
|  | Pregabalin |
|  | Propranol |
|  | Sertraline |
|  | Tramadol |
| Home visits ^1^ | Community psychiatric nurse |

^1^ Source: Resource use questionnaire

^2^ Source: Patient medical records

**Supplementary table 2** - Consolidated Health Economic Evaluation Reporting Standards (CHEERS) checklist

| **Section/item** | **Item No** | **Recommendation** | **Reported on page No/ line No** |
| --- | --- | --- | --- |
| **Title and abstract** | | | |
| Title | 1 | Identify the study as an economic evaluation or use more specific terms such as “cost-effectiveness analysis”, and describe the interventions compared. | page 1, line 1 to 3 |
| Abstract | 2 | Provide a structured summary of objectives, perspective, setting, methods (including study design and inputs), results (including base case and uncertainty analyses), and conclusions. | page 2 |
| **Introduction** | | | |
| Background and objectives | 3 | Provide an explicit statement of the broader context for the study. | page 3, line 1 to  23 |
|  |  | Present the study question and its relevance for health policy or practice decisions. | page 3, line 24 to 30 |
| **Methods** | | | |
| Target population and subgroups | 4 | Describe characteristics of the base case population and subgroups analysed, including why they were chosen. | page 4, line 4 to 9; |
| Setting and location | 5 | State relevant aspects of the system(s) in which the decision(s) need(s) to be made. | page 4, line 6 to 7 |
| Study perspective | 6 | Describe the perspective of the study and relate this to the costs being evaluated. | page 4, line 13 |
| Comparators | 7 | Describe the interventions or strategies being compared and state why they were chosen. | page 4, line 7 to 9 |
| Time horizon | 8 | State the time horizon(s) over which costs and consequences are being evaluated and say why appropriate. | page 4, line 15 to 16 |
| Discount rate | 9 | Report the choice of discount rate(s) used for costs and outcomes and say why appropriate. | page 4, line 16 to 17 |
| Choice of health outcomes | 10 | Describe what outcomes were used as the measure(s) of benefit in the evaluation and their relevance for the type of analysis performed. | page 4, line 20 to 26 |
| Measurement of effectiveness | 11a | *Single study-based estimates:*Describe fully the design features of the single effectiveness study and why the single study was a sufficient source of clinical effectiveness data. | page 4, line 2 to  12 |
|  | 11b | *Synthesis-based estimates*: Describe fully the methods used for identification of included studies and synthesis of clinical effectiveness data. | Not applicable |
| Measurement and valuation of preference based outcomes | 12 | If applicable, describe the population and methods used to elicit preferences for outcomes. | page 4, line 20 to 26 |
| Estimating resources and costs | 13a | *Single study-based economic evaluation:*Describe approaches used to estimate resource use associated with the alternative interventions. Describe primary or secondary research methods for valuing each resource item in terms of its unit cost. Describe any adjustments made to approximate to opportunity costs. | page 4, line 28 to  page 5, line 7 |
|  | 13b | *Model-based economic evaluation:*Describe approaches and data sources used to estimate resource use associated with model health states. Describe primary or secondary research methods for valuing each resource item in terms of its unit cost. Describe any adjustments made to approximate to opportunity costs. | Not applicable |
| Currency, price date, and conversion | 14 | Report the dates of the estimated resource quantities and unit costs. Describe methods for adjusting estimated unit costs to the year of reported costs if necessary. Describe methods for converting costs into a common currency base and the exchange rate. | page 5, line 5 to 7 |
| Choice of model | 15 | Describe and give reasons for the specific type of decision-analytical model used. Providing a figure to show model structure is strongly recommended. | Not applicable |
| Assumptions | 16 | Describe all structural or other assumptions underpinning the decision-analytical model. | Not applicable |
| Analytical methods | 17 | Describe all analytical methods supporting the evaluation. This could include methods for dealing with skewed, missing, or censored data; extrapolation methods; methods for pooling data; approaches to validate or make adjustments (such as half cycle corrections) to a model; and methods for handling population heterogeneity and uncertainty. | page 5, line 9 to  page 6, line 16 |
| **Results** | | | |
| Study parameters | 18 | Report the values, ranges, references, and, if used, probability distributions for all parameters. Report reasons or sources for distributions used to represent uncertainty where appropriate. Providing a table to show the input values is strongly recommended. | Table 2;  Table 3 |
| Incremental costs and outcomes | 19 | For each intervention, report mean values for the main categories of estimated costs and outcomes of interest, as well as mean differences between the comparator groups. If applicable, report incremental cost-effectiveness ratios. | Table 2;  Table 3;  Table 4;  page 7, line 19 to  page 8, line 17 |
| Characterising uncertainty | 20a | *Single study-based economic evaluation:*Describe the effects of sampling uncertainty for the estimated incremental cost and incremental effectiveness parameters, together with the impact of methodological assumptions (such as discount rate, study perspective). | page 8, line 19 to 21 |
|  | 20b | *Model-based economic evaluation:*Describe the effects on the results of uncertainty for all input parameters, and uncertainty related to the structure of the model and assumptions. | Not applicable |
| Characterising heterogeneity | 21 | If applicable, report differences in costs, outcomes, or cost-effectiveness that can be explained by variations between subgroups of patients with different baseline characteristics or other observed variability in effects that are not reducible by more information. | Not applicable |
| **Discussion** | | | |
| Study findings, limitations, generalisability, and current knowledge | 22 | Summarise key study findings and describe how they support the conclusions reached. Discuss limitations and the generalisability of the findings and how the findings fit with current knowledge. | page 9, line 2 to 7 (key findings); page 9, line 8 to 31 (limitations, generalisability); page 10, line 5 to 29 (current knowledge) |
| **Other** | | | |
| Source of funding | 23 | Describe how the study was funded and the role of the funder in the identification, design, conduct, and reporting of the analysis. Describe other non-monetary sources of support. | page 1 |
| Conflicts of interest | 24 | Describe any potential for conflict of interest of study contributors in accordance with journal policy. In the absence of a journal policy, we recommend authors comply with International Committee of Medical Journal Editors recommendations. | Information provided via the submission system |

**Supplementary figure 1 –** PANDA RCT CONSORT flow chart

**Proceeded to consenting and randomisation (n= 671)**

667 consented and completed baseline

4 refused to consent

**Baseline Assessment (n= 667)**

12 refused randomisation

**655 randomised** *

2* did not complete substantial proportion of assessment (excluded from analysis)

**GP suitability checks (n= 1,029)**

893 suitable

110 not suitable

20 GP suitability confirmations not received

6 did not proceed to GP screen

**Patients referred to study at consultation (n= 427)**

**Eligibility checks (n= 1320) of which 893 identified via mail outs and 427 via referral**

671 eligible

113 not eligible

376 declined to take part

160 could not be reached to do telephone screening

**Placebo (n=329)**

1 did not complete PHQ9 or EQ-5D

**Sertraline (n= 324)**

1 did not complete PHQ9 or EQ-5D

**2 week follow-up (n=292)**

292 completed PHQ9 and EQ-5D

8 missed this follow-up

29 withdrew:

22 not interested

4 no time

1 did not want to take drug

2 lost to follow up

**2 week follow-up (n=279)**

277 completed PHQ9 and 288 completed EQ-5D

2 did not complete PHQ9

12 missed this follow-up

33 withdrew:

22 not interested

7 no time

2 did not want to take drug

2 lost to follow-up

**6 week follow-up (n=285)**

285 completed PHQ9 and EQ-5D

1 missed this follow-up

14 withdrew

281completed RUQ

**6 week follow-up (n= 267)**

267 completed PHQ9 and 266 completed EQ-5D

6 missed this follow-up

18 withdrew:

264 completed RUQ

**Identified as potentially eligible via record search (n= 35,719)**

4,074 removed from initial list by GPs

**Sent an invitation letter (n= 31,645)**

1, 029 ‘Yes’ replies

3,399 ‘No’ replies

27,217 no response

**12 week follow-up (n= 265)**

263 completed PHQ9 and 264 completed EQ-5D

2 did not complete PHQ9

3 missed this follow-up

18 withdrew:263 completed RUQ

**12 week follow-up (n=264)**

262 completed PHQ9 and 263 completed EQ-5D

2 did not complete PHQ9

2 missed this follow-up

7 withdrew:262 completed RUQ

**Sample for analysis (n=653)**

**Supplementary figure 2** Total costs and QALYs by severity of depression (according to the CIS-R severity score) and duration of depressive symptoms at baseline

2a Plots total costs against severity

2b Plots QALYs against severity

2c Plots total costs against symptom duration

2d Plots QALYs against symptom duration

**Supplementary Table 3** Incremental net monetary benefit results (complete cases)

|  | £20,000 WTP | £30,000 WTP |
| --- | --- | --- |
|  | INMB (95% CI) | INMB (95% CI) |
| Model 1 (n=379) |  |  |
| Sertraline | £104.06 (£-182.19 to £390.31) | £144.8 (£-228.41 to £518.01) |
| Severity |  |  |
| Low | Reference | Reference |
| Moderate | £-60.81 (£-347.26 to £225.65) | £-102.89 (£-476.36 to £270.59) |
| High | £-69.86 (£-383.59 to £243.86) | £-127.55 (£-536.58 to £281.48) |
| Sertraline * severity |  |  |
| Low | Reference | Reference |
| Moderate | £-6.78 (£-382.78 to £369.22) | £12.34 (£-477.89 to £502.56) |
| High | £22.77 (£-310.99 to £356.53) | £29.96 (£-405.19 to £465.11) |
| Model 2 (n=379) |  |  |
| Sertraline | £121.85 (£-29.71 to £273.4) | £176.59 (£-20.99 to £374.18) |
| Duration (>2 years) | £72.93 (£-118.23 to £264.1) | £66.02 (£-183.2 to £315.24) |
| Sertraline * Duration (>2 years) | £-24.58 (£-300.24 to £251.07) | £-41.03 (£-400.4 to £318.34) |
| Model 3 (n=380) |  |  |
| Sertraline vs placebo | £122.46 (£-3.02 to £247.95) | £174.1 (£10.55 to £337.65) |

Abbreviations: CI, confidence interval; INMB, incremental net monetary benefit; WTP, willingness-to-pay

Model 1: Interaction model between sertraline and baseline severity, adjusted for severity, duration, baseline utility and site practice

Model 2: Interaction model between sertraline and duration, adjusted for duration, severity, baseline utility and site practice

Model 3: Non-interaction model comparing sertraline vs placebo, adjusted for baseline utility and site practice

**Supplementary Table 4** Incremental net monetary benefit results (excluding secondary costs - imputed)

|  | £20,000 WTP | £30,000 WTP |
| --- | --- | --- |
|  | INMB (95% CI) | INMB (95% CI) |
| Model 1 (n=650) |  |  |
| Sertraline | £129.73 (£-61.11 to £320.57) | £167.02 (£-91.52 to £425.57) |
| Severity |  |  |
| Low | Reference | Reference |
| Moderate | £7.53 (£-191.21 to £206.28) | £-15.19 (£-278.47 to £248.08) |
| High | £-57.14 (£-316.57 to £202.3) | £-94.3 (£-439.71 to £251.11) |
| Sertraline * severity |  |  |
| Low | Reference | Reference |
| Moderate | £-48.72 (£-307.73 to £210.28) | £-25.67 (£-376.28 to £324.95) |
| High | £4.59 (£-228.53 to £237.71) | £11.61 (£-315.14 to £338.36) |
| Model 2 (n=650) |  |  |
| Sertraline | £124 (£10.1 to £237.91) | £174.55 (£17.66 to £331.43) |
| Duration (>2 years) | £-0.02 (£-137.87 to £137.83) | £-15.92 (£-212.96 to £181.12) |
| Sertraline * Duration (>2 years) | £-14.32 (£-232.14 to £203.5) | £-24.57 (£-338.48 to £289.34) |
| Model 3 (n=653) |  |  |
| Sertraline vs placebo | £122.61 (£28.56 to £216.66) | £171.89 (£40.21 to £303.57) |

Abbreviations: CI, confidence interval; INMB, incremental net monetary benefit; QALY, quality-adjusted life year

Model 1: Interaction model between sertraline and baseline severity, adjusted for severity, duration, baseline utility and site practice

Model 2: Interaction model between sertraline and duration, adjusted for duration, severity, baseline utility and site practice

Model 3: Non-interaction model comparing sertraline vs placebo, adjusted for baseline utility and site practice

**Supplementary Table 5** Incremental net monetary benefit results (excluding costs not thought to be associated with mental health care - imputed)

|  | £20,000 WTP | £30,000 WTP |
| --- | --- | --- |
|  | INMB (95% CI) | INMB (95% CI) |
| Model 1 (n=650) |  |  |
| Sertraline | £94.03 (£-71.39 to £259.45) | £131.32 (£-109.05 to £371.7) |
| Severity |  |  |
| Low | Reference | Reference |
| Moderate | £-50.71 (£-217.58 to £116.15) | £-73.44 (£-315.11 to £168.23) |
| High | £-106.14 (£-332.65 to £120.37) | £-143.3 (£-465.73 to £179.13) |
| Sertraline * severity |  |  |
| Low | Reference | Reference |
| Moderate | £42.77 (£-177.13 to £262.66) | £65.82 (£-255.87 to £387.52) |
| High | £-55.63 (£-357.16 to £245.9) | £25.02 (£-289.48 to £339.52) |
| Model 2 (n=650) |  |  |
| Sertraline | £129.02 (£25.96 to £232.09) | £179.57 (£31.19 to £327.95) |
| Duration (>2 years) | £-5.18 (£-138.86 to £128.51) | £-21.08 (£-214.83 to £172.67) |
| Sertraline * Duration (>2 years) | £-42.56 (£-249.96 to £164.83) | £-52.81 (£-357.34 to £251.71) |
| Model 3 (n=653) |  |  |
| Sertraline vs placebo | £118.59 (£30.8 to £206.37) | £167.87 (£41.09 to £294.66) |

Abbreviations: CI, confidence interval; INMB, incremental net monetary benefit; QALY, quality-adjusted life year

Model 1: Interaction model between sertraline and baseline severity, adjusted for severity, duration, baseline utility and site practice

Model 2: Interaction model between sertraline and duration, adjusted for duration, severity, baseline utility and site practice

Model 3: Non-interaction model comparing sertraline vs placebo, adjusted for baseline utility and site practice
